# New Methods to Study the Behavior of Molecularly Imprinted Polymers in Aprotic Solvents

**DOI:** 10.3390/polym10091015

**Published:** 2018-09-12

**Authors:** Anett Nagy-Szakolczai, Zsanett Dorkó, Blanka Tóth, George Horvai

**Affiliations:** 1 Department of Inorganic and Analytical Chemistry, Budapest University of Technology and Economics, Szent Gellert ter 4., H-1111 Budapest, Hungary; nagyszakolczai.anett@gmail.com (A.N.-S.); dorko.zsanett@gmail.com (Z.D.); 2 MTA-BME Research Group of Technical Analytical Chemistry, Szent Gellert ter 4., H-1111 Budapest, Hungary

**Keywords:** molecular imprinting, propranolol, beta blocker, conductivity, displacement, adsorption isotherm, ion exchange, Langmuir

## Abstract

This work presents three new experimental methods for studying molecular imprinting. The electric conductivity measurements of the pre-polymerization mixture of amine templates in an aprotic solvent provide evidence of ionic dissociation of the pre-polymerization complexes. The displacement measurement of the template propranolol from its molecularly imprinted polymer (MIP) using a quaternary ammonium ion in toluene, shows that this MIP behaves as an ion exchanger even in a non-polar solvent. The same experiment also shows that template binding to the MIP from toluene involves ionic interaction. The third experimental method introduced here serves to study the models of template binding on MIPs. To this end the binding isotherm of propranolol (PR) has been measured on a polymer mixture consisting of non-imprinted control polymer (NIP) and a stronger binding acidic polymer, respectively. All three methods are suitable for studying several other imprinting systems.

## 1. Introduction

Molecular imprinting is a vigorous field of research [1,2,3,4,5,6,7,8,9]. Each year more than a thousand new papers are registered in the molecularly imprinted polymer (MIP) database [10]. MIPs are also commercially available. There are many potential applications of MIPs, including analytical ones. MIPs for small molecules and macromolecules, like proteins, are made in great variety [1,2,3,4,5,6,7,8,9,10]. MIPs for microorganisms have also been described.

Despite the great progress in molecular imprinting over the last few decades, there is still little information available on the structure of the binding sites of MIPs and about the exact nature of the binding interactions. Most MIPs are solids or soft gels, and analytical techniques to directly study the structure of individual binding sites and of individual binding events in such matrices are rare [11]. Thus, information about the binding sites is obtained using mainly indirect methods, like adsorption isotherm measurements, analysis of the pre-polymerization mixture or using computer modelling [11,12,13,14,15,16,17,18,19,20,21,22,23,24,25,26,27,28,29,30,31,32,33,34,35,36,37].

Studies of MIP adsorption isotherms [11] have led some investigators to believe that many MIPs have two kinds of binding sites [12,13]. One type of site binds the template or another target molecule selectively and strongly, while the other type is weak and nearly unselective. The concentration of the strong sites is usually estimated to be much lower than that of the weak sites. For example, Andersson [14] investigated the isotherm of a propranolol imprinted polymer that was made using a functional monomer methacrylic acid (MAA), in the aprotic solvent toluene. He found that the concentration of strong binding sites was merely 2 mmol/kg, while that of the weak sites was 38 mmol/kg. Both types of sites are generally assumed to follow the Langmuir adsorption isotherm. Other isotherm studies [15,16,17] proposed further models. Affinity distribution analyses led to continuous distribution of sites, from strong and selective sites present in small numbers, to weaker and less selective sites present in far greater densities [11]. Apart from measuring isotherms, isothermal titration calorimetry (ITC) can be used to study the heat effects of adsorption on MIPs [18,19,20,21].

The study of pre-polymerization mixtures is an alternative approach for the better understanding of imprinting. The binding between the template and the monomer(s), which already exists in the pre-polymerization mixture, is expected to constitute an important part of the binding interactions with the solidified polymer. This has been one of the motivations for spectroscopic and computational studies of the pre-polymerization mixtures [22,23,24,25,26,27,28,29,30,31,32,33,34,35,36,37]. NMR investigation of the pre-polymerization mixture has been particularly informative concerning the stoichiometries and stabilities of pre-polymerization complexes [22,23,24,25,26]. When NMR measurement continued into the first phase of polymerization, until gelation occurred, the interactions between the template and functional monomer were found to remain unchanged [27]. It was concluded that the interactions observed in the pre-polymerization mixture are also retained in MIP. Pre-polymerization mixtures of protein templates may be studied also by differential scanning fluorimetry (DSF) [28]. Computer modelling of the pre-polymerization mixture [25,29,30,31,32,33,34,35,36] and of the imprinting process [37] has also revealed interesting relationships regarding the interactions between all components in the pre-polymerization mixture and has contributed to the rational development of MIPs.

Despite these achievements, understanding of imprinting has been found very recently to be far from satisfactory [37]. This view has been supported also by our recent paper [38] which has shown that the effect of imprinting on selectivity can be different from typical expectations.

In the present paper, three novel experimental methods are introduced with the goal of better understanding of the non-covalent imprinting phenomenon. The first method consists of measuring and interpreting the specific conductivity of an aprotic pre-polymerization mixture, possibly for the first time. Using this method, evidence can be obtained about the presence of ions in the aprotic pre-polymerization mixture. The second method involves a new kind of displacement experiment on MIPs. The amine template, propranolol, can be displaced from the MIP by quaternary ammonium ions in toluene solution. This is an unexpected effect for the aprotic and non-polar medium used here. This directly proves the ion exchange behavior of the MIP, and the ionic binding of the originally neutral template in a non-polar medium. The third method introduces adsorption experiments on polymer mixtures as a tool to study binding site distribution on MIPs. This shows, for the first time, by direct experiment, that template adsorption on MIP may arise as the sum of adsorption on sites of the non-imprinted control polymer (NIP) and stronger binding sites, respectively.

All three methods are being used here to investigate the non-covalent imprinting of amine type templates with an acidic functional monomer in aprotic solvents. The proposed methods may be used, however, with many other imprinting systems.

## 2. Materials and Methods

### 2.1. Materials

Propranolol hydrochloride, methacrylic acid, dibenzylamine (DBA), ethylene glycol dimethacrylate (EDMA), tetrabutylammonium hydroxide solution (40% in water) and inhibitor remover (Aldrich 306312) were purchased from Sigma-Aldrich (St. Louis, MO, USA). Sodium hydroxide (NaOH), sodium dihydrogen phosphate monohydrate (NaH_2_PO_4_·H_2_O), tetrabutylammonium bromide (TBABr) and azobisisobutyronitrile (AIBN) were obtained from Fluka (Buchs, Switzerland). Acetonitrile (HPLC grade), methanol, toluene and methyl tert-butyl ether were ordered from Merck (Darmstadt, Germany). Water was purified with a Milli Q Direct 8 system (Millipore, Burlington, MA, USA). HCl was purchased from Riedel-De Haën (Seelze, Germany). The weak cation exchanger, Strata-X-CW, was obtained as a free sample from Phenomenex (Macclesfield, UK). Its binding capacity was 0.76 mol/kg according to the manufacturer.

The structure of the used compounds can be found in the Supplementary Materials (Figure S1).

### 2.2. Instrumentation

The following instruments were used in this study: Series 200 HPLC (Perkin Elmer, Waltham, MA, USA), Purospher RP18-e (125 mm × 3 mm, 5 μm, Merck) reversed phase column, Orion Star A212 Conductivity Meter with Orion 013005 MD electrode, Orion model 290A pH meter with Orion 910600 Thermo electrode (Thermo Fisher Scientific, Waltham, MA, USA), Grant-bio PTR-35 multirotator (Grant Instruments, Cambridge, UK), Minispin centrifuge (Eppendorf, Hamburg, Germany), TurboVap LV Concentration Evaporator (Zymark, Hopkinton, MA, USA), and a magnetic stirrer.

### 2.3. Synthesis of the Propranolol MIP

Propranolol MIP was prepared, as described previously [39], using a modification of Andersson’s method [14]. Prior to use, methacrylic acid and ethylene glycol dimethacrylate were purified using an Aldrich 306312 inhibitor remover column, according to the manufacturer’s instructions [40]. Propranolol hydrochloride was transformed into a free base before use by means of neutralization with 0.2 M NaOH solution, followed by extraction with methyl tert-butyl ether. The pre-polymerization mixture was prepared in a glass vial. Its composition with respect to the template and monomers was same as that of a polymer prepared by Andersson and denoted by him as polymer A [14]. Briefly, the molar ratio of template (PR): functional monomer (MAA): crosslinker (EDMA) was 1:8:40. The solvent (ACN)/total monomer volume ratio was 1.33 and the amount of initiator was 0.7 mol% of the total double bond amount of the monomers (further details can be found in Table S1 of the Supplementary Materials). The mixture was purged with argon for 5 min, tightly sealed with a PTFE septum cap and was placed under a UV source (366 nm) for 24 h at room temperature. The formed bulk polymer was crushed and ground in a mortar. The corresponding non-imprinted polymer (NIP) was prepared in the same manner but the template was omitted. NIP was thoroughly washed with methanol. MIP was washed several times with 0.01 M HCl solution in methanol–water (1:1) to remove the template, and then it was washed thoroughly with methanol. After washing, the polymers were dried overnight at room temperature.

### 2.4. Equilibrium Binding Measurements

Equilibrium binding measurements of propranolol on different polymers were carried out at room temperature (25.0 ± 3.0 °C). Polymers were weighed into polypropylene microtubes and the solution of analyte was pipetted into the tube. The adsorption isotherms were measured by varying the initial concentration of the analyte solutions. In the competitive binding measurements, the tetrabutylammonium bromide concentration was 0.005 M in toluene, while the propranolol concentration was varied. In earlier works, it was found that 30 min was enough to reach equilibrium [39,41]. Thus, after mixing for 30 min, the samples were centrifuged. Except for measurements in toluene, the supernatant was directly diluted with the HPLC eluent and injected into the HPLC system to quantify the unbound propranolol concentration. In the case of binding experiments in toluene, the supernatant was evaporated and the residual was dissolved in the HPLC eluent.

Detailed information concerning initial concentrations and the phase ratio used in these experiments can be found in the Supplementary Materials (Table S2).

For each test on MIP, leaching of the template from the polymer particles was checked to be negligible by a blank measurement without template addition. Reproducibility of the adsorption measurements was checked by duplicating some of the measurements.

Measurement of the propranolol concentration was accomplished by HPLC on a reversed phase column (Merck LiChroCART Purospher RP-18 endcapped 5 μm (125 mm × 3.0 mm)). The eluent flow rate was 0.6 mL/min, the injection volume was 10 μL and the wavelength of detection was 215 nm. The isocratic eluent was a phosphate buffer ACN mixture in 70:30 v/v ratio. The phosphate buffer was 10 mM in NaH_2_PO_4_, with the pH adjusted to 3.0 by H_3_PO_4_. The retention factor of propranolol was 2.7.

The equilibrium adsorbed concentration (*q*) of the analyte was calculated according to Equation (1).

(1) $$\;q=\left(c_{0}-c\right)\cdot F\;$$

where *c*_0_ and *c* are the initial and the equilibrium solution concentrations [M] of the analyte, respectively, *F* is the phase ratio (solution [mL] to polymer [g] ratio). The equilibrium adsorbed concentration (*q*) was plotted against the equilibrium solution concentration (*c*) of the respective compounds in a log *c*–log *q* plot [39].

### 2.5. Conductometric Experiments

The conductometric electrode was calibrated with the following solutions: 692 ppm NaCl, Orion 011007 (1413 μS/cm) (Thermo Fisher Scientific) and 0.00056 M KCl, 51302453 (84 μS/cm) (Mettler Toledo, Columbus, OH, USA). The measurements were carried out at room temperature (25.5 ± 1.0 °C).

## 3. Results

Three novel experimental methods for studying non-covalent molecular imprinting are presented here. The first one involves conductivity measurements of pre-polymerization mixtures of MIPs, while the other two are adsorption experiments on MIP and on other polymers.

### 3.1. Ionic Conductivity of the Pre-Polymerization Mixture

Conductivity measurements of pre-polymerization mixtures were made to demonstrate the presence of ions in these mixtures. Pre-polymerization mixtures of MIPs for propranolol and di–benzylamine, respectively, have been set up as during MIP preparation, with 1:1, 1:4 and 1:8 template to functional monomer (MAA) ratio, respectively. Their specific conductivity was measured. For comparison, the specific conductivity of solutions of two tetrabutylammonium salts in the pre-polymerization medium were also measured. All investigated solution compositions are shown in the Supplementary Materials (Table S3). The measured specific conductivities are presented in Table 1.

The initiator was omitted from the pre-polymerization mixtures to avoid any polymerization and because its role is secondary here.

### 3.2. Displacement of the Template from the MIP by a Quaternary Ammonium Cation

Ionic displacement experiments have been made to demonstrate the ion exchange behavior of the propranolol imprinted polymer. MIP was prepared using propranolol as the template, and MAA as the functional monomer, with a 1:8 template to functional monomer ratio. The crosslinker was EDMA, the porogen was acetonitrile. The composition of the pre-polymerization mixture for the MIP was the same as the one used for the conductometric measurements with 1:8 ratio amine: MAA, only the initiator was added to it.

**Figure 1:**
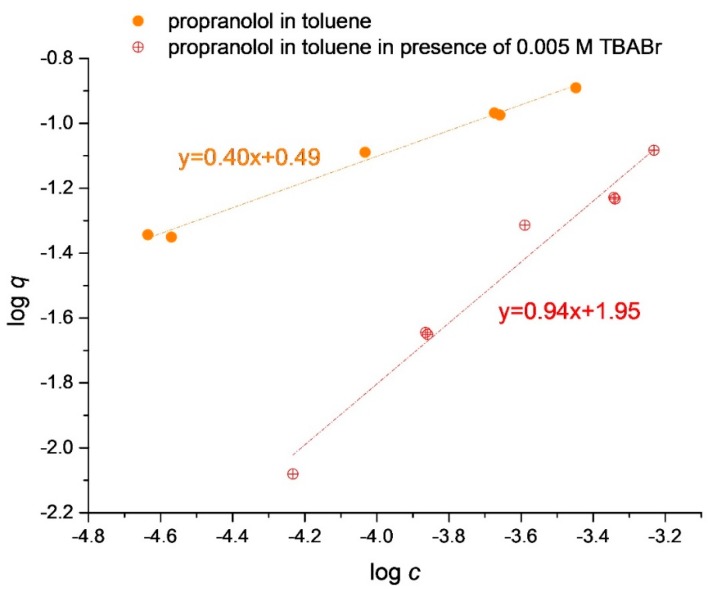
Adsorption isotherms of propranolol on the propranolol imprinted polymer (MIP) measured in toluene.

The adsorption isotherm of the template, propranolol, was measured on MIP in toluene at room temperature, in the absence and in the presence of 0.005 M tetrabutylammonium bromide, respectively (Figure 1). The four points measured in duplicate, as shown in Figure 1, show that reproducibility of the measurements was satisfactory. Note that, in duplicate measurements, both *c* and *q* differ, thus the usual characterization by the standard deviation of the dependent variable, *q*, would not be appropriate.

### 3.3. Emulation of the MIP’s Isotherm by a Mixture of the NIP with Another Carboxylic Polymer

To simulate and thereby understand the template adsorption behavior of propranolol MIP, a mixture of its non-imprinted control polymer (NIP) with the commercial carboxylic polymer Strata-X-CW was made, and the adsorption isotherm of propranolol on this polymer mixture was measured. The mass mixing ratio of Strata-X-CW to the NIP was 1 to 4.4.

**Figure 2:**
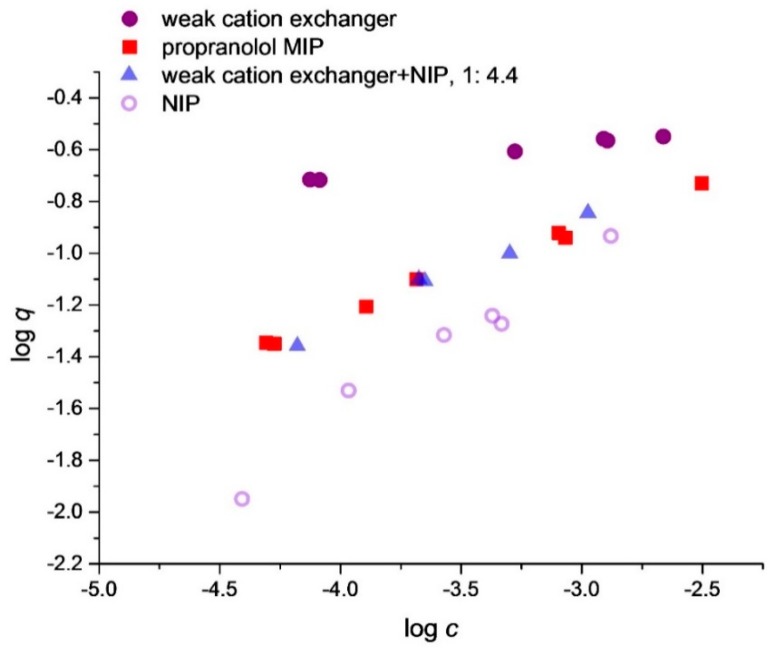
Adsorption isotherm of propranolol on different polymers and on a polymer mixture, measured in acetonitrile.

For comparison, the adsorption isotherms of propranolol were also measured on propranolol MIP and on the individual components of the polymer mixture, i.e., on Strata-X-CW and on NIP, respectively. All isotherms were determined in acetonitrile. Figure 2 shows the four isotherms as a series of measurement points. The close agreement of the six duplicates is visible. Isotherm functions are intentionally not fitted to the measured data because no isotherm function is used in the discussion.

## 4. Discussion

In this paper, three novel methods are presented for studying molecular imprinting. From the vast range of molecular imprinting systems, only one, but a rather important one, is considered: The imprinting for amine templates using the non-covalent method, using an acidic functional monomer, MAA. The main template used here, propranolol, has been called the work-horse template for fundamental investigations of the molecular imprinting process [42].

### 4.1. Ionic Conductivity of the Pre-Polymerization Mixture

The first of the three novel methods consists of measuring the conductivity of the pre-polymerization mixture. Simple as it is, this method apparently has not been used yet to gain information about the species present in the pre-polymerization mixture. One reason for this may be that one does not expect anything conspicuous from conductivity measurements, either in aqueous or in non-polar pre-polymerization media. In aqueous media the amine template and the acidic functional monomer form fully dissociated salts, so that the pre-polymerization mixture would have high specific conductivity. In non-polar media no free ions are expected to be present, thus the conductivity should be very low. In the present work, however, the porogen is acetonitrile, a non-protic, but polar, solvent. The reaction of weak bases with weak acids in acetonitrile leads to characteristic changes in the conductivity of the solution [43,44]. The chemical phenomena behind these conductivity changes are much more complex than in aqueous solutions. This subject has been thoroughly studied [43,44], but, as far as we know, the results of these studies have not been used yet for investigating MIP pre-polymerization mixtures using conductivity measurements.

The essence of the known facts about weak base–weak acid interactions in acetonitrile is that, complexes of various stoichiometries may be formed and the propensity of each complex for ionic dissociation is different. The typical complexes are of $B{\left(HA\right)}_{n}$ stoichiometry, where B is the base and HA is the acid, while n is a small integer. These complexes may be hydrogen bonded or ion pairs, or both, so that the H-bonded form and the ion pair form are in equilibrium with each other [45]. The complexes may also undergo ionic dissociation. This leads typically to a $BH^{+}$ cation and ${\left[{\left(HA\right)}_{n-1}A\right]}^{-}$ anion. Interestingly, the degree of ionic dissociation of the $B{\left(HA\right)}_{2}\;$ complex may be higher than that of the BHA, i.e., 1:1 complex.

The medium of the pre-polymerization mixtures in this work is a mixture of acetonitrile with the crosslinker EDMA. The presence of EDMA makes the medium less polar than pure acetonitrile. The conductivity data presented in line four of Table 1 show that ionization occurs in the pre-polymerization mixture with a 1:8 ratio of the template propranolol and functional monomer MAA, respectively. The first line of Table 1 shows that, in the presence of the propranolol base alone, the conductivity was very low. The same is true if only the acid MAA is present at a 0.42 M concentration, which is its concentration in the pre-polymerization mixture (Table 1). These observations show that the conductivity of the pre-polymerization mixture cannot be attributed to the ionic dissociation of either propranolol or MAA. Thus, it must be due to the ionic dissociation of propranolol–MAA complexes of the above-described types.

This observation of free ions in a pre-polymerization mixture is a novelty. It shows that, in the pre-polymerization mixture, one or more complexes, which are formed between the template and the functional monomer, MAA, can dissociate into ions. It may be assumed that the complexes found in the pre-polymerization mixture became fixed in the MIP during polymerization [27], so we can expect that when the MIP rebinds the template from acetonitrile, ionic species will also be formed, and the MIP will behave as an ion exchanger. This conclusion is further corroborated by the other experiments introduced in this paper.

Before turning to the other experiments, it is interesting to note that the observed ionization is not unique for propranolol. As Table 1 shows, the 1:8 pre-polymerization mixture of dibenzylamine has a similar conductivity as the 1:8 pre-polymerization mixture of propranolol. One can also see in Table 1 that the 1:4 and 1:8 pre-polymerization mixtures of both propranolol and dibenzylamine conduct much better than their respective equimolar mixtures. In view of the literature [43,44], where similar observations have been made in other systems, it is not surprising that the four-fold or eight-fold excess of MAA against the amine template in the pre-polymerization mixtures increases the conductivity compared to the 1:1 mixture. Further work should elucidate how this observation is related to the known effect that excess MAA often results in better MIPs.

It is interesting to estimate the degree of ionization of the pre-polymerization complexes. In Section 5 of the Supplementary Materials, such an estimation is presented.

### 4.2. Displacement of the Template from the MIP by a Quaternary Ammonium Cation

The second novel method relates to studying the MIPs themselves. When a MIP contacts a solution of its template, some of the template is adsorbed on the MIP. This is called rebinding. Rebinding may occur from various media, and the medium has great effect on the extent and chemistry of rebinding. If the medium is an aqueous buffer, the rebinding of an amine template on an acidic MIP is likely to occur by a combination of ion exchange and hydrophobic effects [12]. In non-polar media, like toluene, rebinding is expected to occur by hydrogen bonding and van der Waals forces, while ionic binding in form of ion pairs has been judged unlikely [46]. The conductivity experiments above had shown that in a polar medium containing acetonitrile the pre-polymerization complexes can ionize, and this made it likely that adsorption of an amine template from acetonitrile on the MIP occurs at least partly in the ion pair form. By going one step further one may ask if in a non-polar medium like toluene the adsorption of an amine on the acidic MIP may also occur in ion pair form, despite opposite earlier expectations [46]. A novel kind of competitive binding experiment has been introduced to clarify the above statement.

When a MIP is immersed into a mixture of its template and of another amine, dissolved in a non-polar solvent, the second amine will compete with the template for the adsorption sites of MIP. This is natural if rebinding occurs via hydrogen bonding, because both amines may form hydrogen bond with the acid groups of the MIP. However, if a quaternary ammonium salt is used instead of an amine competitor, then replacement of the template appears unlikely because the quaternary ammonium ions are not likely to form a hydrogen bond.

Against this expectation we have found that a quaternary ammonium salt, tetrabutylammonium bromide can displace the amine template, propranolol, from its MIP in the non-polar medium of toluene. The displacement is made evident by the large downward shift of the propranolol adsorption isotherm in the presence of tetrabutylammonium bromide, as seen in Figure 1. In the leftmost part of Figure 1, where the relative excess of the quaternary ion is the largest, the vertical distance between the logarithmic isotherms is about 1.0, so that approximately 90% of propranolol is displaced from the MIP by tetrabutylammonium. Such a displacement is only possible if propranolol is displaced from the MIP as propranololium cation (Figure S1) and not as the neutral amine, because, otherwise, electroneutrality would not be preserved.

The competition experiment with tetrabutylammonium shows that the MAA-based MIP can behave as an ion exchanger even in toluene. The precondition for this is that a neutral amine must be adsorbed first on the MIP. This amine, the template in our experiments, neutralizes the carboxylic groups and turns the MIP into a cation exchanger. Competition by the quaternary ammonium ion releases the amine template in its cationic form, not in the amine form, in which it had been adsorbed. These results show that the binding of propranolol from toluene occurs at least partly in ionic form, i.e., the carboxylic group of the MIP donates a hydrogen ion to the template in the course of binding.

### 4.3. Emulation of the MIP’s Isotherm by a Mixture of the NIP with Another Carboxylic Polymer

The third novel experiment introduced in this paper consists of comparing the isotherms of a MIP with the isotherms of some other, non-imprinted polymers, and with the isotherm of a mixture of the latter.

Since the binding sites of MIPs are very difficult to be studied directly, much of the information about these sites has been obtained by indirect methods. One of the most frequently used indirect methods is the measurement of the template’s adsorption isotherm on the MIP. The interpretation of these isotherms requires, however, assumptions which are difficult to prove. One typical assumption is [12,13,47,48] that the MIP has two or more kinds of binding sites and all of these sites behave according to the Langmuir adsorption equation. It is assumed that non-covalent imprinting creates some strong and selective binding sites for the template, but there are also many other binding sites on the MIP which are weakly and non-selectively binding the template [11]. Experimental support for these ideas comes mainly from isotherm measurements which are interpreted by mathematical decomposition into Langmuir sites. The isotherm measurements need to be extremely accurate and precise if useful data are to be extracted from them about the assumed, but unproved, Langmuirian binding sites [49]. Most of the published isotherm data are, however, of a lower precision than would be optimal [49].

Here, we have taken a different approach to the study of adsorption isotherms. We anticipated that due to the excess of MAA used in making the MIP, only part of the carboxylic groups of the MIP can be assigned to the imprinted binding sites, while the remaining carboxylic groups will behave as the carboxylic groups of NIP. In agreement with all other authors, we assumed also that the imprinted sites bind the template stronger than the non-imprinted ones, at least on average. Thus, our goal has been to see if a polymer, which is known to have two types of binding sites, part of these sites being identical to the sites of the NIP, and the remaining sites binding the template much more strongly than NIP, will indeed produce an isotherm which is nearly the same as the MIP’s isotherm. Synthesizing such a polymer might be difficult, but one can easily mix the NIP with another, more strongly binding non-imprinted polymer. Suitable porous polymers with carboxylic groups for the strong adsorption of amines are commercially available. One of these, Strata-X-CW from Phenomenex, appeared, on the basis of preliminary investigations, to be suited to be included in the two-polymer mixture as the strongly binding polymer. Note that Strata-X-CW is not an imprinted polymer.

Figure 2 shows the adsorption isotherms of propranolol on the propranolol MIP, on the NIP, on Strata-X-CW and on a 4.4 to 1 w/w mixture of the NIP and Strata-X-CW, respectively. Propranolol binding on the NIP is the lowest, and on Strata-X-CW it is at its highest. The difference between these two polymers is mainly in their respective binding strength, because their carboxyl group contents differ only by about thirty percent (NIP 1.0 mol/kg, Strata-X-CW 0.76 mol/kg), while their propranolol binding differs by up to one order of magnitude, depending on the solution concentration.

Figure 2 shows also, that the isotherm of the polymer mixture nearly overlaps with the isotherm of the MIP. This is a direct evidence showing that by replacing some of the NIP sites with stronger sites, one can obtain a polymer with a template binding isotherm just like that of the MIP.

The 4.4:1 proportion of the NIP to Strata-X-CW was found by preliminary calculations from the two polymers’ and the MIP’s individual isotherms.

These results are significant because the model used here does not assume Langmuir type sites or any mathematical adsorption equation. Rather, the only assumption is that there are two groups of sites on the MIP, one group being essentially the same kind as on the NIP, and the other group stronger than the NIP sites. This method therefore opens a new route for studying adsorption on MIPs.

## 5. Conclusions

This paper demonstrates the usefulness of three novel methods for studying MIPs. Conductivity experiments carried out with the pre-polymerization mixture of basic templates, and using the functional monomer MAA, have shown for the first time that the pre-polymerization complexes are capable of ionic dissociation even in aprotic media. This result is also useful information for molecular dynamics simulations of pre-polymerization mixtures, because the molecular dynamics of neutral molecules, if not combined with quantum chemistry, cannot lead to the appearance of ions. Future detailed studies of the conductivity of pre-polymerization solutions may also reveal more information about the detailed composition of the complexes between the template and functional monomer.

The competitive displacement of an amine template, propranolol, from its MIP by a quaternary ammonium ion, observed in the non-polar solvent toluene, provided evidence of the ion-exchange behavior of MIP in a non-polar environment. It has also shown that the neutral amine is bound by the MIP in the ionic form, at least partly. This agrees with the expectations derived from the conductivity experiments of the pre-polymerization mixture.

The complex problem of interpreting the template binding isotherm of MIPs in terms of the binding site composition has been studied by a conceptually novel experiment. A mixture of NIP with another, but much stronger, binding carboxylic polymer was used to simulate the MIP isotherm. When mixing the two polymers in an appropriate ratio, the resulting mixture’s adsorption isotherm became virtually identical with the isotherm on the MIP, confirming the applicability of the new approach. 

All three methods presented here are expected to be useful for studying many other imprinting systems in the future.

## Figures and Tables

**Table 1:** The specific conductivities of different pre-polymerization mixtures and control solutions.

	**Amine: MAA molar ratio**	**Specific conductivity (μS/cm)**
propranolol prepolym. mixtures	1:0	0.6
	1:1	9.5
	1:4	44.5
	1:8	66.8
DBA prepolym. mixtures	1:0	0.5
	1:1	14.2
	1:4	52.8
	1:8	81.7
	**Concentration (M)**	**Specific conductivity (μS/cm)**
MAA	0.42	2.2
TBABr	0.01	598
TBAMAA	0.01	489

## Supplementary Materials

The following are available online at http://www.mdpi.com/2073-4360/10/9/1015/s1, Figure S1: Structures of compounds, Table S1: The composition of the PR MIP, Table S2: Initial concentrations and phase ratios, F, used in adsorption experiments, Table S3: Composition of the systems used in the conductometric measurements.
