# Supplementary material for: New Methods to Study the Behavior of Molecularly Imprinted Polymers in Aprotic Solvents
Source: Polymers (Basel). 2018 Sep 12;10(9):1015. doi: 10.3390/polym10091015 (PMC6403568; doi:10.3390/polym10091015)
Supplement: Supplementary file 1 [file polymers-10-01015-s001.pdf]

Supplementary Material:

New methods to study the behavior of molecularly imprinted polymers in aprotic solvents

Anett Nagy-Szakolczai[1], Zsanett Dorkó [1,2], Blanka Tóth[1*] and George Horvai[1,2,*]

[1] Department of Inorganic and Analytical Chemistry, Budapest University of Technology and Economics, Szent Gellert ter 4., H-1111 Budapest, Hungary; nagyszakolczai.anett@gmail.com (A.N.-S.), dorko.zsanett@gmail.com (Z.D.)

[2] MTA-BME Research Group of Technical Analytical Chemistry, Szent Gellert ter 4., H-1111 Budapest, Hungary

* Correspondence: tblanka@mail.bme.hu (B.T.), george.horvai@mail.bme.hu (G.H.); Tel.: +36-1-4631480 (G.H.)

1. Structure of compounds

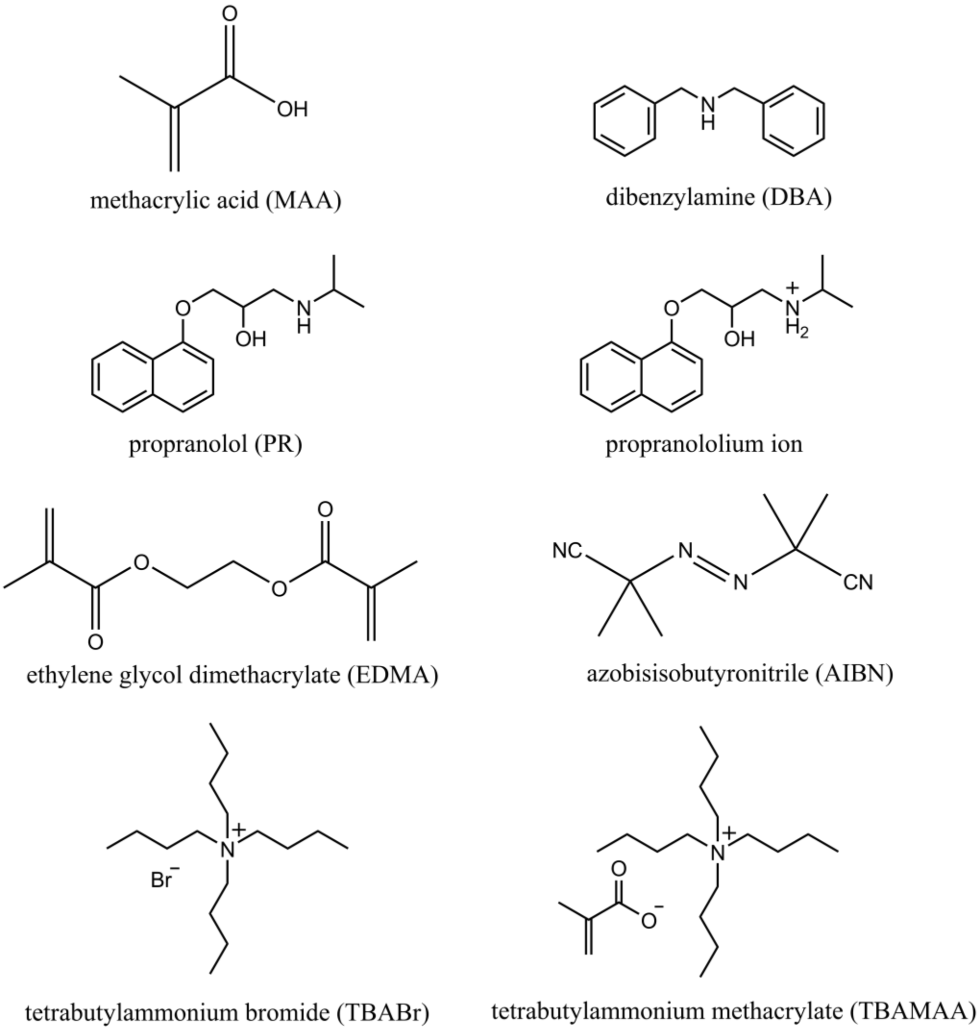

Figure S1. Structures of compounds.

2. Polymer preparation

Table S1. The composition of the PR MIP.

Compound	Added amount	
PR	55.0 mg	0.212 mmol
MAA	0.143 mL	1.69 mmol
EDMA	1.591 mL	8.43 mmol
ACN	2.310 mL	-
AIBN	21.3 mg	0.130 mmol

3. Equilibrium binding measurements

Table S2. Initial concentrations and phase ratios, F, used in adsorption experiments.

Polymer	*F*	medium	initial concentration of PR (M)
PR MIP	100	ACN	$5 \cdot 10^{-4} - 5 \cdot 10^{-3}$
Phenomenex Strata-X-CW	100	ACN	$2 \cdot 10^{-3} - 5 \cdot 10^{-3}$
Phenomenex Strata-X-CW:NIP, 1:4.4	100	ACN	$5 \cdot 10^{-4} - 2.5 \cdot 10^{-3}$
NIP	100	ACN	$1.5 \cdot 10^{-4} - 2.5 \cdot 10^{-3}$
PR MIP (competition measurement)	200	toluene	$10^{-4} - 10^{-3}$

4. Compositions used in the conductometric measurements

Table S3. Composition of the systems used in the conductometric measurements.

System	Amine: MAA molar ratio	amount of amine or salt	amount of MAA	amount of EDMA	amount of ACN
		mmol	*mmol*	*mmol*	*mL*
propranolol prepolymerization mixtures	1:0	1.10	-	43.85	12.0
	1:1	1.10	1.10	43.85	12.0
	1:4	1.10	4.40	43.85	12.0
	1:8	1.10	8.81	43.85	12.0
DBA prepolymerization mixtures	1:0	1.10	-	43.85	12.0
	1:1	1.10	1.10	43.85	12.0
	1:4	1.10	4.40	43.85	12.0
	1:8	1.10	8.80	43.85	12.0
MAA in prepolym. medium	-	-	8.81	43.85	12.0
TBABr in prepolym. medium	-	0.201	-	43.85	12.0
TBAMAA in prepolym. medium	-	0.201	-	43.85	12.0

5. Estimation of the degree of dissociation in the prepolymerization mixture of the propranolol MIP

In the main part of the paper, it has been deduced from the conductivity of the prepolymerization mixture of the propranolol MIP that the prepolymerization complexes dissociate ionically. The degree of dissociation cannot be determined with certainty without further investigations into the composition and distribution of complexes being present. A rough estimate may be obtained, however, by comparing the measured specific conductivities of the prepolymerization mixtures with those of salts which are likely to ionize at a high extent in the prepolymerization medium. Table 1 in the paper shows that two such salts, tetrabutylammonium methacrylate and tetrabutylammonium bromide, both at 0.01 M concentration, have much higher conductivity than the prepolymerization mixtures have in the same medium. Thus one can estimate, that the ion concentrations in the prepolymerization mixtures are much lower than 0.01 M, and therefore the degree of dissociation of the prepolymerization complexes, the concentration of which is limited by the template concentration of 0.053 M, is likely to be below ten percent.
